# Dr. James E. Reeves

**Published:** 1896-02

**Authors:** 


### DR. JAMES E. REEVES.

THE death of Dr. James E. Reeves, of Chattanooga, January 4th, removes one of the most prominent and distinguished of Southern physicians and sanitarians. Dr. Reeves was a Virginian by birth, and the first thirty years of his professional life were spent in West Virginia. He was the chief organizer of the West Virginia Medical Society in 1867, and subsequently become president of the same. He was the author of the law creating the State Board of Health of West Virginia, of which he was made a member and secretary for five years. He was one of the founders of the American Public Health Association, of which he was president in 1885. He was also vice-president of the section in public and international hygiene of the International Medical Congress in Washington in 1887. About eight years ago Dr. Reeves removed to Chattanooga. For a number of years he had been especially interested in normal and pathological histology and was the author of several valuable papers in this department of medical science, and also of a systematic text-book which was published about one year ago. This book was kindly received by critics, and gave evidence of both literary scholarship and scientific attainments. One of Dr. Reeves's prominent characteristics was his avowed enmity to quackery and chicanery in all their shapes and wherever found. He was honest and ethical in the highest degree, and his professional acts and status were above reproach. One of the last acts of his life was his arraignment of the Amick Consumption Cure Company. How he exposed this quack concern, and how he was sued by the company for damages, and how the courts decided in his favor on every article of the allegations, have already been told in these pages. The profession has lost a worthy and venerable representative in Dr. Reeves. We are sorry that he is gone and regret there are not others like him.

